# Microscopic pulmonary tumor embolism from adenocarcinoma of the prostate

**DOI:** 10.1002/iju5.12159

**Published:** 2020-06-18

**Authors:** Tatsuya Hattori, Yosuke Ikegami, Nayuka Matsuyama, Takashi Hamakawa, Tetsuji Maruyama, Aya Naiki‐Ito, Takahiro Yasui

**Affiliations:** ^1^ Department of Urology Nagoya City East Medical Center Nagoya Japan; ^2^ Department of Education and Research Center for Advanced Medicine Nagoya City University Graduate School of Medical Sciences Nagoya Japan; ^3^ Department of Experimental Pathology and Tumor Biology Nagoya City University Graduate School of Medical Sciences Nagoya Japan; ^4^ Department of Nephro‐urology Nagoya City University Graduate School of Medical Sciences Nagoya Japan

**Keywords:** androgen receptor, cadherin, microscopic pulmonary tumor embolism, prostate cancer, pulmonary hypertension

## Abstract

**Introduction:**

Microscopic pulmonary tumor embolisms from prostate cancer are extremely rare. In this case of prostate cancer, microscopic pulmonary tumor embolism developed during androgen deprivation therapy.

**Case presentation:**

A 56‐year‐old man was diagnosed with prostate cancer and underwent androgen deprivation therapy. Three months after starting treatment, he noticed shortness of breath and developed acute progressive dyspnea. He was diagnosed with pulmonary hypertension; however, the cause was not found. His dyspnea was progressive and he died 40 days after the onset of symptoms. Autopsy proved that the cause of pulmonary hypertension was microscopic pulmonary tumor emboli from prostate cancer. Furthermore, histology revealed differences in the androgen receptors in the prostate and emboli, with significantly greater Ki‐67 expression in the emboli than in the prostate.

**Conclusion:**

Prostate cancer proliferated in the pulmonary artery after hematogenous metastasis, caused vascular occlusion, and formed microscopic pulmonary tumor embolisms.

**Abbreviations & Acronyms**
ALP: alkaline phosphatase
AR: androgen receptor
FDP: fibrinogen degradation products
LDH: lactate dehydrogenase
PSA: prostate‐specific antigen
PSMA: prostate‐specific membrane antigen

**Keynote message**

Microscopic pulmonary tumor embolism from prostate cancer is rare. In this case a tumor developed in a lung vessel and occluded the pulmonary artery. The subacute progression of the case supported the finding of hematogenous metastasis.

## Introduction

Microscopic pulmonary tumor embolisms from prostate cancer are extremely rare. We report a case of microscopic pulmonary tumor embolism caused by prostate cancer, which was found at autopsy.

## Case presentation

**Fig. 1:**
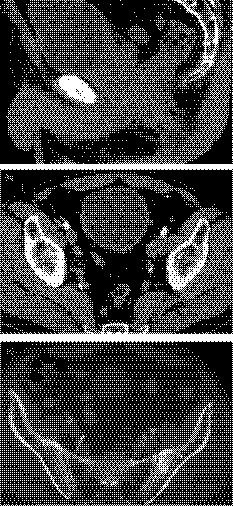
Pelvic computed tomography at diagnosis. (a) Sagittal view shows that the tumor has invaded the bladder. (b, c) Horizontal views show metastases to the bilateral lymph nodes and iliac bone.

A 56‐year‐old Japanese man with a serum PSA level of 24.6 ng/mL was diagnosed with stage IV prostate cancer in May 2018 after prostatic biopsy. The Gleason score was 5 + 5, and computed tomography showed that prostate cancer had invaded the bladder (Fig. 1a), with metastases to the proximal bilateral lymph nodes (Fig. 1b) and left iliac bone (Fig. 1c). Staging was T4N1M1b, according to the UICC TNM Classification of Malignant Tumours, 8th edition. His LDH was 298 U/L and ALP was 229 U/L. He underwent androgen deprivation therapy with abiraterone (1000 mg), leuprorelin (22.5 mg), and prednisolone (5 mg). One month later, his PSA decreased to 16.1 ng/mL, LDH was 252 U/L, and ALP was 327 U/L. Three months later, he noticed shortness of breath on exertion, and his symptoms worsened in October 2018. He was admitted to another hospital and diagnosed with pulmonary hypertension, but the cause of the pulmonary hypertension was not found. He was sent to our hospital for further examination after 3 days.

On admission, his blood pressure was 108/64 mmHg, heart rate 110/min, oxygen saturation 93% with 6 L of O_2_, and respiration rate 30/min. The average pulmonary artery pressure was 28.6 mmHg in the catheterization performed at the previous hospital. Arterial blood gas analysis showed a pH of 7.473, PaO_2_ 76.9 Torr, PaCO_2_ 32.7 Torr, and HCO_3_‐ 23.6 mmol/L. His white cell count was 5370/μL, platelets 28 000 μL, and FDP level was 30.9 μg/mL. His PSA level was 22.5 ng/mL, LDH was 2529 U/L, and ALP was 2425 U/L. The antinuclear antibody was negative.

A decrease in platelets and an increase in FDP were observed, suggesting the presence of disseminated intravascular coagulation. Contrast‐enhanced computed tomography of the thorax revealed slightly enlarged pulmonary vessels with no parenchymal lesions.

The patient was diagnosed with pulmonary thrombosis. Despite treatment with oxygen inhalation, intravenous heparin, phosphodiesterase inhibitor, and urokinase, he died of respiratory failure 2 days after admission.

**Fig. 2:**
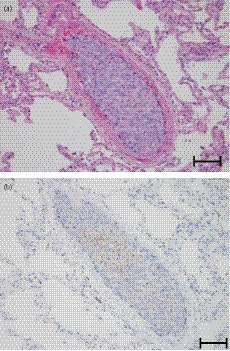
PSMA staining of the cell mass. (a) Hematoxylin and eosin staining and (b) PSMA staining of the pulmonary artery mass. Scale bar is 200 μm.

Autopsy confirmed pulmonary hemorrhage. The prostate was replaced with tumor tissue, but there was no dissemination of poorly differentiated prostate cancer to the diffuse pulmonary arteries. There was no lymph node metastasis. Microscopic findings revealed diffuse pulmonary occlusion due to a cell mass. The cell mass was positive for PSMA (Fig. 2), and the cause of death was determined to be pulmonary artery occlusion due to prostate cancer. In the prostate, there was no vascular invasion with a volume that could become an embolus.

**Fig. 3:**
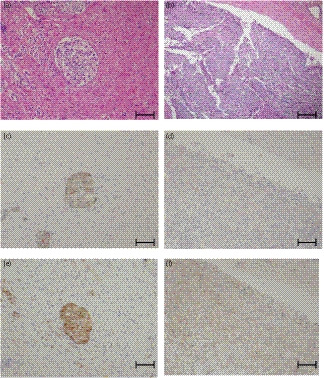
E‐cadherin and N‐cadherin staining. Hematoxylin and eosin staining of (a) the prostate and (b) pulmonary artery; E‐cadherin staining of (c) the prostate and (d) pulmonary artery; N‐cadherin staining of (e) the prostate and (f) pulmonary artery. Scale bar is 200μm.

**Fig. 4:**
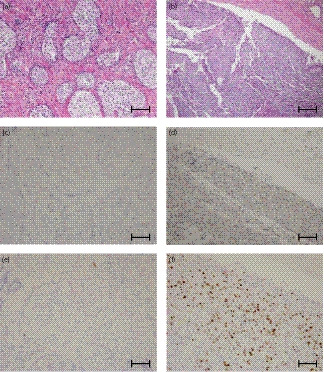
AR and Ki‐67 staining. Hematoxylin and eosin staining of (a) the prostate and (b) pulmonary artery; AR staining of (c) the prostate and (d) pulmonary artery; Ki‐67 staining of (e) the prostate and (f) pulmonary artery. Scale bar is 200 μm.

Epithelial‐mesenchymal transition is involved in the formation of tumor emboli, and we stained for its expression markers; E‐cadherin and N‐cadherin. E‐cadherin was suppressed and N‐cadherin was upregulated in both the prostate and the pulmonary artery (Fig. 3). Despite androgen deprivation therapy, the patient’s symptoms were progressing and heterogenesis was suspected in the lesion. When AR staining was performed, AR expression was not observed in the prostate, but was observed in the pulmonary artery. In order to evaluate cell proliferation, we stained with Ki‐67. The pulmonary tissue was stained 39% and the prostate was stained 3% (Fig. 4).

## Discussion

This unusual case presents a microscopic pulmonary tumor embolism that developed during treatment for advanced prostate cancer. Kane *et al*. reviewed 1085 autopsies of solid malignant tumors and found microscopic pulmonary tumor embolism associated with carcinoma in 2.4% of the cases.^1^ It was considered to be fatal in 1% of the autopsy studies.^1^ Reports of such embolisms in prostate cancer are rare, and there were only six well‐documented cases of death due to microscopic pulmonary tumor embolism secondary to prostate cancer.^1^, ^2^, ^3^, ^4^, ^5^ Microscopic pulmonary tumor embolisms occur when a tumor mass directly occludes the pulmonary artery or when a tumor grows within the pulmonary artery. In the first case, a malignant tumor forms a mass in a blood vessel, and a part of it separates and occludes the vessel. In this mechanism, shortness of breath progresses rapidly. Histopathological findings show that the expression of E‐cadherin is involved in forming a cell mass.^6^ On the other hand, with occlusion due to intravascular growth of a tumor, the tumor grows at the metastasis destination and occludes the blood vessel, and respiratory failure proceeds subacutely.^7^, ^8^ In addition, N‐cadherin expression is involved in hematogenous metastasis of tumors.^9^

In this case, the progression of dyspnea was relatively slow. At autopsy, there was no vascular invasion with a volume that could become an embolus, and immunochemistry supported this hypothesis of hematogenous metastasis. In the embolism, E‐cadherin was suppressed, and N‐cadherin was upregulated. In addition, AR expression was observed in the embolism, but not in the prostate, suggesting that the prostate cancer had become castration resistant. Based on these facts, it was inferred that the pulmonary tumor embolism was caused by prostatic cancer that had metastasized to the blood, proliferated in the pulmonary blood vessels, and occluded the pulmonary artery over 40 days.

Treatment of lung tumor embolism requires treatment of the underlying disease.^2^ In this case, treatment with abiraterone and prednisolone ultimately failed. If the PSA follow‐up interval was shortened, changes in PSA might have been detected earlier and chemotherapy might have been initiated. In addition, the appearance of dyspnea during exertion may suggest consideration of treatment changes.

## Conclusion

This rare case revealed a microscopic pulmonary tumor embolism that developed during prostate cancer treatment. This was thought to have developed due to hematogenous metastasis and intravascular growth of the tumor.

## Ethical approval

This article was approved by the Nagoya City East Medical Center Institutional Review Board, approval number 19‐04‐368.

## Consent for publication

Written informed consent was obtained from the patient’s family for publication of this case report and accompanying images.

## Conflict of interest

The authors declare no conflict of interest.
